# Wider translations and rotations in posterior-stabilised mobile-bearing total knee arthroplasty compared to fixed-bearing both implanted with mechanical alignment: a dynamic RSA study

**DOI:** 10.1007/s00167-023-07541-6

**Published:** 2023-08-24

**Authors:** Raffaele Zinno, Domenico Alesi, Stefano Di Paolo, Nicola Pizza, Stefano Zaffagnini, Giulio Maria Marcheggiani Muccioli, Laura Bragonzoni

**Affiliations:** 1 Dipartimento Di Scienze Per La Qualità Della Vita QUVI, University of Bologna, Corso D’Augusto 237, 47921 Rimini, RN Italy; 2 2nd Orthopaedic and Traumatologic Clinic, IRCCS, Istituto Ortopedico Rizzoli, Via G.B. Pupilli 1, 40136 Bologna, BO Italy; 3 Dipartimento di Scienze Biomediche e Neuromotorie DIBINEM, University of Bologna, Via San Vitale, 40125 Bologna, BO Italy

**Keywords:** Radiostereometric analysis, Posterior stabilised, Fixed bearing, Mobile bearing, Medial pivot, Mechanical alignment

## Abstract

**Purpose:**

The purpose of this study was to investigate the in vivo kinematics of the same femoral design mechanically aligned posterior-stabilised (PS) total knee arthroplasty (TKA) with either fixed-bearing (FB) or mobile-bearing (MB) inlay, implanted by the same surgeon, using model-based dynamic radiostereometric analysis (RSA). The hypothesis of the present study was that the MB design would show wider axial rotation than the FB design, without affecting the clinical outcomes.

**Materials and methods:**

A cohort of 21 non-randomised patients (21 DePuy Attune PS-FB) was evaluated by dynamic RSA analysis at a minimum 9-month follow-up, while performing differently demanding daily living activities such as sit to stand (STS) and deep knee lunge (DKL). Kinematic data were compared with those of a cohort of 22 patients implanted with the same prosthetic design but with MB inlay. Anterior–posterior (AP) translations, varus–valgus (VV) and internal–external (IE) rotations of the femoral component with respect to the tibial baseplate were investigated. Translation of medial and lateral compartment was analysed using the low point method according to Freeman et al. Questionnaires to calculate objective and subjective clinical scores were administered preoperatively and during follow-up visit by the same investigator.

**Results:**

The FB TKA design showed lower AP translation during STS (6.8 ± 3.3 mm in FB vs 9.9 ± 3.7 mm in MB, p = 0.006*), lower VV rotation (1.9 ± 0.8° in FB vs 5.3 ± 3.3° in MB, p = 0.005) and lower IE rotation (2.8 ± 1.1° in FB vs 9.5 ± 4.3° in MB, p = 0.001) during DKL than the mobile-bearing TKA design. Posterior-stabilised FB group showed significant lower translation of the low point of the medial compartment than the MB group (p = 0.008). The percentage of patients performing medial pivot in the FB group was higher compared to MB group in the examined motor tasks. No significant differences in post-operative range of motion (117° ± 16° for FB group and 124° ± 13° for MB group) and in clinical outcomes emerged between the two cohort.

**Conclusions:**

The FB and MB designs differed in AP translations, VV rotations and IE rotations of the femoral component with respect to the tibial component in STS and DKL. Furthermore, FB cohort reported a significant higher percentage of medial pivot with respect to MB cohort. Despite this, no differences in clinical outcomes were detected between groups. Both designs showed stable kinematics and represent a viable option in primary TKA.

**Level of evidence:**

Prospective cohort study, II.

## Introduction

A significant percentage of patients report no longer feeling confident using the operated knee in activities of daily living after total knee arthroplasty (TKA) [6, 9]. This could be attributable to non-physiological kinematics of the prosthetic knee [40]. To improve the performance of TKA, new designs have been introduced over the years, and the literature shows as these can influence clinical outcomes [18, 27]. However, the influence kinematics on knee function is not established.

For example, mobile-bearing (MB) TKA designs with femur rotating together with the polyethylene have shown a wide range of axial rotation between the insert and tibial plateau. This should reduce the shear forces on the articular surface and, thus, the polyethylene wear [14, 23]. However, no clear evidence of kinematic difference between fixed-bearing (FB) and MB insert prostheses has been reported so far [28, 37, 38].

An accurate methodology comparing the same TKA femoral design with two different inlay configurations could therefore provide precious insights on the actual kinematical differences between MB and FB avoiding design-related confounding factors.

Thus, the aim of this study was to evaluate the in vivo kinematics of a mechanically aligned specific posterior-stabilised TKA design, implanted either with a FB and MB inlay, with an accurate technique such as model-based dynamic radiostereometric analysis (RSA). The hypothesis of the present study was that the MB design would show wider axial rotation than the FB design, without affecting the clinical outcomes.

## Materials and methods

Twenty-one patients who underwent TKA with cemented posterior-stabilised FB design (Attune™ Knee System, De Puy Synthes, Johnson & Johnson, Warsaw, IN, USA) were enrolled in this prospective study after providing an informed consent. Surgeries were performed by the same senior surgeon and adjusted mechanical alignment [33] adopting measured resection with subsequent releases was used for all the patients. This cohort of 21 patients with FB design was compared with a cohort of 22 patients who underwent TKA with a similar prosthetic design with MB insert, who already participated in a study with the same set-up and similar follow-up time [26]. The implants investigated have the same PS femoral component with multiple radii of curvature in the sagittal plane and differ in the design of the tibial plateau and the insert. The FB tibial tray has a central locking design for the polyethylene, while the metal back of the MB design is a flat and highly polished cobalt chromium surface with very low surface roughness, allowing freedom of the polyethylene insert to rotate around a central polyethylene post. The inclusion/exclusion criteria are reported in Table 1.

**Table 1:** Inclusion/Exclusion criteria description

	Inclusion criteria	Exclusion criteria
1	Age range: 50–85 years	Previous corrective osteotomy on the affected lower limb
2	Severe radiographic primary osteoarthritis (Kellgren–Lawrence grade 3 and grade 4)	Post-traumatic arthritis
3	Patients scheduled for a primary total knee arthroplasty	Severe pre-operative varus–valgus deformity (Hip Knee Ankle angle > 10°)
4		Body Mass Index > 40 kg/m2
5		Rheumatoid arthritis
6		Chronic inflammatory joint diseases
7		Patients with a pre-pathological abnormal gait (amputated, neuromuscular disorders, poliomyelitis, developmental dysplasia of the hip)
8		Severe ankle osteoarthritis (Kellgren–Lawrence grade 3 and grade 4)
9		Severe hip osteoarthritis (Kellgren–Lawrence grade 3 and grade 4)
10		Previous total hip or ankle replacement
11		Unwillingness to take part in this study
12		Incomplete clinical or kinematical assessment
13		Inability to perform the motor tasks

In this study, Patient-Reported Outcome Measures (PROMs) were collected prior to the surgical procedure and again at a minimum of 9 months post-surgery, while demographic data were exclusively obtained before surgery. Furthermore, the kinematic evaluation was conducted at a minimum of 9 months after the surgical intervention only.

The RSA set-up was already described in previous publications from the same study group [1, 26, 30]. In brief, the RSA device is composed by 2 radiographs tubes (RTM 101HS, IAE, Milan, Italy) and 2 digital flat panels (PIXIUM RF4343, Thales Electron Devices SA, Vèlizy-Villacoublay, France). The two beamlines were positioned perpendicular to each other and synchronised to acquire two contemporary set of images with a frame rate of 8 frame per second.

All patients were evaluated by model-based dynamic RSA, while performing low and high demanding motor tasks. Specifically, the two examined motor tasks were:

- Sit to stand (STS): the patients stood up from a 40 cm high radiolucent chair without support;
- Deep knee lunge (DKL): the patient performed a lunge on the operated leg up to the maximum allowed flexion, subsequently returning to the upright position.

The AP translations and VV and IE rotations were described as the displacement of the femoral component relative to that of the tibial component. The displacement was considered relatively to the centroid of the 3D CAD model of each prosthetic component as provided by the manufacturer. The dynamic RSA's overall accuracy in model positioning and orientation, expressed as “trueness ± precision”, was found to be sub-millimetric, with 0.2 mm ± 0.5 mm for positioning and 0.3° ± 0.2° for orientation [1, 5, 30].

The femur and tibia references systems were described in previous studies. Briefly, the flexion angle was assessed along the *X*-axis (positive rotation: flexion). The anterior–posterior translations and the varus–valgus rotation were assessed along the *Y*-axis (positive translation: anterior; positive rotation: varus). The internal–external rotations were assessed along the *Z*-axis (positive rotation: internal) [26]. The kinematic quantitative data for each patient were calculated using the Grood and Suntay decomposition method [17].

To analyse the presence of medial pivot, independent movement of the medial and lateral condyles was used according to the low point technique described by Freeman et al.[15]. The “pivoting ratio”, defined as AP translation range of medial and lateral compartment of each patient, was compared according to the formula:

$$Pivoting\,ratio=\frac{A-\mathrm{B}}{\mathrm{A}+\mathrm{B}},$$

where “*A*” was the lateral low point translation range and “*B*” is the medial low point translation range. A “pivoting ratio” was assessed within (− 1, 1) for each task. In all tasks with ratio between 0.1 and 1, prostheses were considered to perform consistent medial pivot. A ratio between 0.1 and − 0.1 was considered as cylindrical rollback, while a ratio between − 0.1 and − 1 was considered lateral pivot according to current literature [2, 8, 12, 15, 24].

Questionnaires to calculate clinical scores (Visual Analogue Scale, Western Ontario and McMaster University, Knee injury and Osteoarthritis Outcome Score, Oxford, clinical and functional Knee Society Score) were administered by the same investigator at each follow-up visit.

This study obtained the approval from Institutional Review Board (IRB) of the IRCCS Rizzoli Orthopaedic Institute (ID: 0035594 October 22, 2015).

### Statistical analysis

Statistical analysis was performed with MATLAB (The MathWorks, Natick, Massachusetts, USA). The differences in kinematic variables between MB and FB groups were assessed using the Student’s *t* test for one-dimensional analysis in the Statistical Parametric Mapping (SPM-1D) software [29]. The matched pair *t* test was used to assess the statistical differences between medial and lateral low point ranges of the femoral components as well as for the pre-/post-operative range of motion clinically evaluated. The two-tailed *t* test was used to compare either medial or lateral low point femoral compartments between the two TKA designs. Student’s *t* test for unpaired samples was used for parametric quantitative variables to compare demographic data, follow-up time and clinical scores between the two groups. Differences were considered statistically significant for *p* < 0.05.

## Results

Demographic data of the two cohort of patients are reported in Table 2. No statistical difference was detected between the two groups, apart from the follow-up time, which extended the described 9 months in both groups. After an interval of 9 months, the patients sensation of pain should coincide with those of the general population, also if a considerable number of patients still have problems in performing strenuous activities [20].

**Table 2:** Demographic and radiographic data of the two examined cohort

	FB	MB	*P*-value
N°	21	22	
Age	71.6 ± 6.7	74.5 ± 7.7	n.s
FU time	11.7 ± 2.1	17.2 ± 7.0	0.001*
Gender	11 males/10 females	12 Males/10 females	
Leg	14 right/7 Left	7 right/15 left	
**Phenotype**	6 Valgus/15 Varus	2 Valgus/20 Varus	
**HKA pre**	3.9 ± 10.5	6.8 ± 5.7	n.s
**HKA post**	1.2 ± 3.1	1.8 ± 3.0	n.s
**MPTA**	87.0 ± 4.8	86.2 ± 3.3	n.s
**LDFA**	88.4 ± 3.1	88.8 ± 1.8	n.s
**JLCA**	4.7 ± 1.7	4.5 ± 1.1	n.s

Positive HKA value indicates varus

*FB* fixed bearing, *MB* mobile bearing

*Statistically significant values

All patients were able to perform the motor tasks in the MB group, while 2 patients did not perform DKL in the fixed-bearing group because they did not feel safe performing that high demanding motor task.

**Fig. 1:**
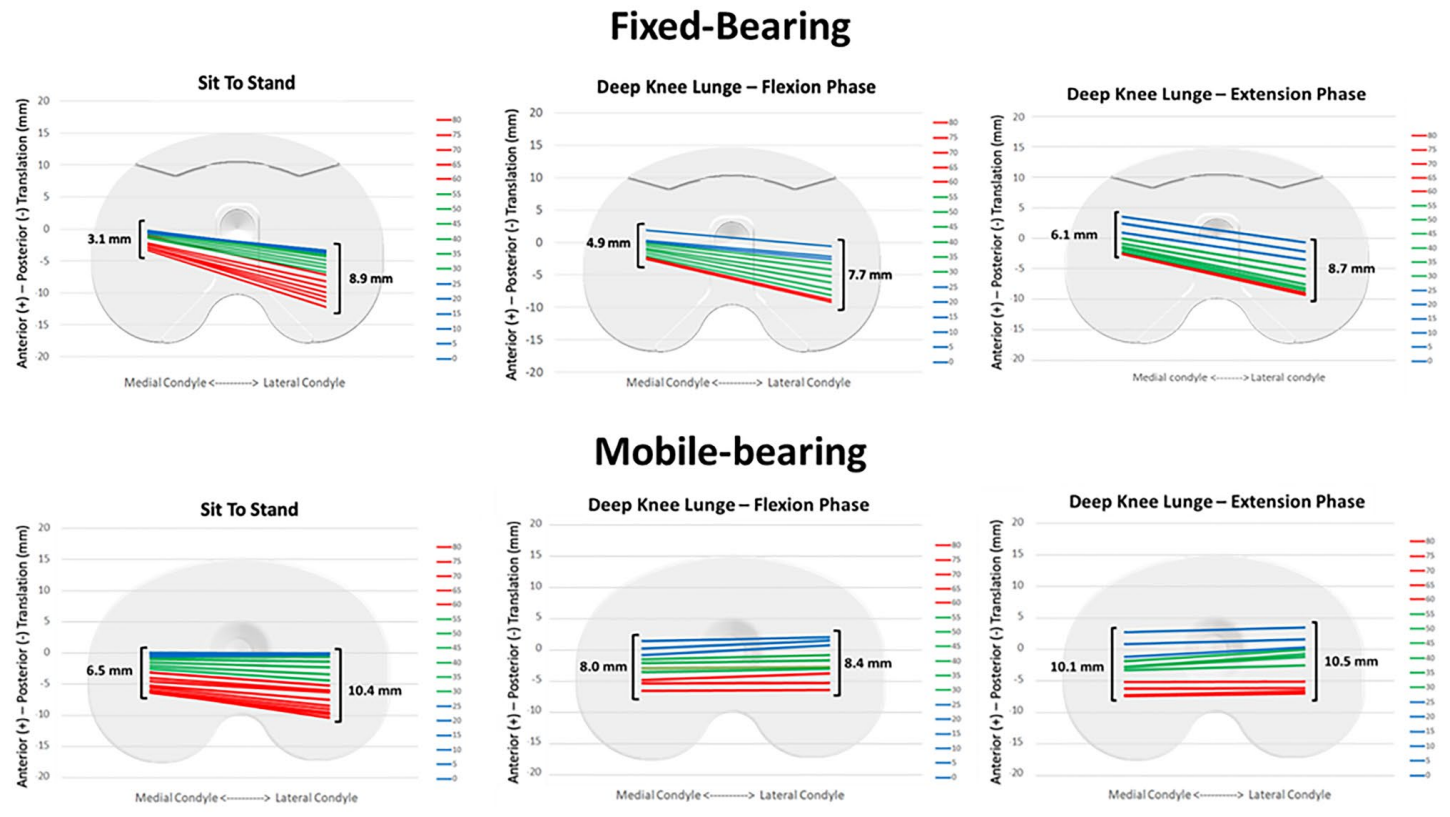
Low point kinematics of fixed-bearing and mobile-bearing designs during the examined motor task. A significant greater translation of the lateral compartment compared to medial one was detected in the two examined motor task. *FB* fixed bearing, *MB* mobile bearing

**Fig. 2:**
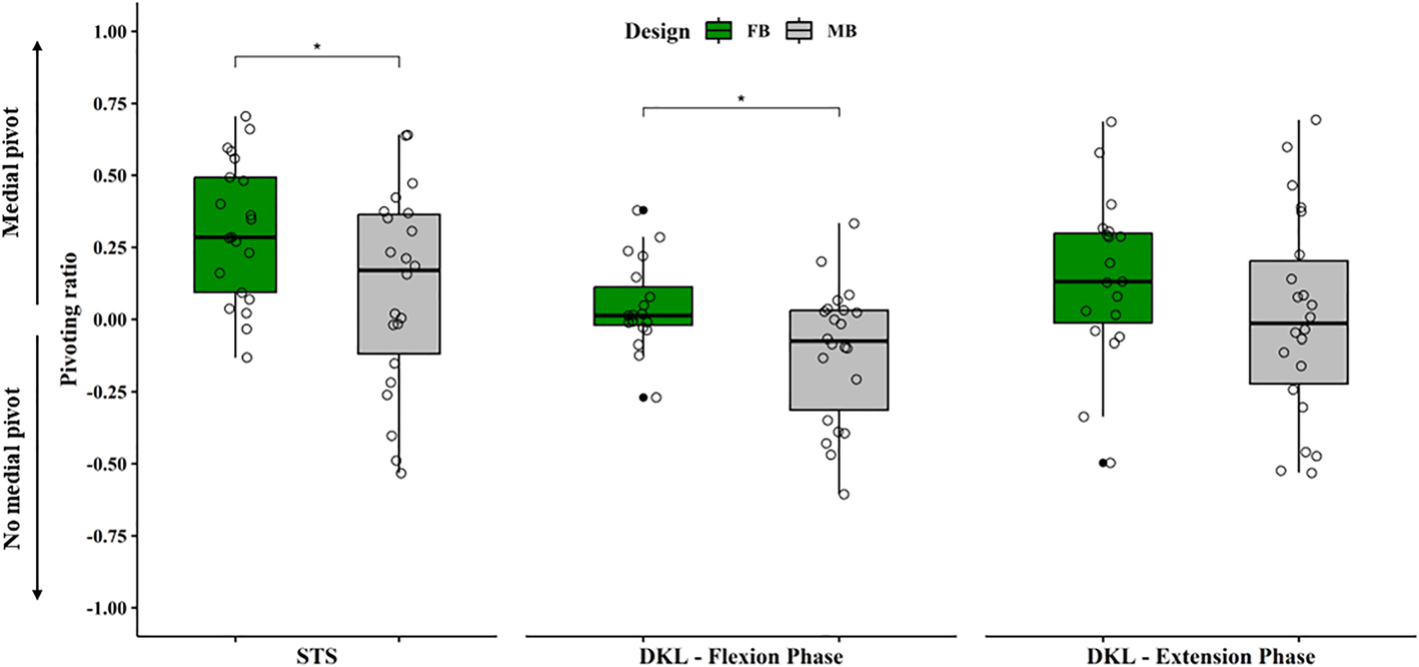
Boxplots showing the pivot ratio (ratio between antero-posterior translation of lateral and medial compartment) for sit to stand (left), flexion phase of deep knee lunge (middle) and extension phase of deep knee lunge (right). The green box represents the fixed-bearing group and the grey box represents the mobile-bearing group. Small circles represent the single pivot ratio values. Values greater than 0.1 were considered medial pivot pattern. *FB* fixed bearing, *MB* mobile bearing, *STS* sit to stand, *DKL Flx* deep knee lunge flexion, *DKL Ext* deep knee lunge extension

Statistically significant differences have been found between the two investigated designs in post-operative AP translations and in VV and IE rotations of femoral relative to tibial component, respectively, in STS and DKL (Table 3). Posterior-stabilised FB implant showed significantly greater translation of the low point of the lateral compartment with respect to that of the medial compartment during either STS or flexion phase of DKL, while posterior-stabilised MB implant only during STS movement (Table 4, Fig. 1). Furthermore, posterior-stabilised FB group showed a significant lower translation of the low point of the medial compartment than the MB group, while no significant difference was shown for the lateral compartment (Table 4). A significantly higher rate of patients with medial pivot in the FB group than in the MB group was detected in STS and flexion phase of DKL motor tasks (*p* < 0.05), while the extension phase of the DKL did not show statistically significant differences (*p* > 0.05) (Table 5 and Fig. 2).

**Table 3:** Anterior–posterior translation, varus–valgus and internal–external rotation of fixed-bearing and mobile-bearing design during the two examined motor tasks

	Sit to stand (Mean range ± SD)			*Deep *knee lunge (Mean range ± SD)		
	FB	MB	*P*-value	FB	MB	*P*-value
Anterior–posterior translation (mm)	6.8 ± 3.3	9.9 ± 3.7	0.006*	7.5 ± 4.8	9.3 ± 6.6	n.s
Varus–valgus rotation (°)	2.6 ± 0.9	3.2 ± 1.3	n.s	1.9 ± 0.8	2.8 ± 1.1	0.005*
Internal–external rotation (°)	7.3 ± 3.8	9.8 ± 4.8	n.s	5.3 ± 3.3	9.5 ± 4.3	0.001*

*FB* fixed bearing, *MB* mobile bearing, *SD* standard deviation

*Statistically significant values

**Table 4:** Anterior–posterior translation of the low point of both designs during the examined motor tasks

	Sit to stand (Mean range ± SD)			Deep knee lunge (ext) (Mean range ± SD)			Deep knee lunge (flx) (Mean range ± SD)		
	FB	MB	*P*-value	FB	MB	*P*-value	FB	MB	*P*-value
Medial compartment AP translation (mm)	3.1 ± 3.3	6.5 ± 4.5	0.008*	6.1 ± 2.9	10.1 ± 4.5	0.002*	4.4 ± 2.9	8.0 ± 4.5	0.004*
Lateral compartment AP translation (mm)	8.9 ± 3.7	10.4 ± 4.5	n.s	8.7 ± 4.1	10.5 ± 4.5	n.s	8.6 ± 4.1	8.4 ± 4.5	n.s
*P*-value	< 0.001*	0.006*		n.s	n.s		< 0.001*	n.s	

*FB* fixed bearing, *MB* mobile bearing, *Ext* extension, *Flx* flexion, *SD* standard deviation

*Statistically significant values

**Table 5:** Percentage of patients showing medial pivot, lateral pivot and cylindrical roll back kinematics during the examined motor tasks

	STS		DKL flex		DKL ext	
	FB	MB	FB	MB	FB	MB
Medial pivot	15	12	16	10	11	7
Lateral pivot	1	6	1	6	2	8
Cylindrical roll back	5	4	2	6	6	7
% Medial pivot	71%	55%	84%	45%	58%	32%

*STS* sit to stand, *DKL Flx* deep knee lunge flexion, *DKL Ext* deep knee lunge extension, *FB* fixed bearing, *MB* mobile bearing

Post-operative range of motion (ROM), clinically evaluated by the same operator using a goniometer, was 117° ± 16° for FB group and 124° ± 13° for MB group. This difference was not statistically significant.

Post-operative clinical outcomes of the two investigated cohort are reported in Table 6. Despite two patients in the FB group were not able to perform DKL because they did not feel safe, no significant differences were reported comparing post-operative clinical outcomes between the two groups. Clinical outcomes of the patients who did not perform the DKL were in line with the rest of the FB cohort (Table 7).

**Table 6:** Average post-operative clinical outcomes of the two investigated cohort showing no significant difference between the two groups

	FB (*n* = 21)	MB (*n* = 22)	*T* test *P*-value
VAS	1.9 ± 2.5	1.5 ± 1.8	n.s
Womac	84.1 ± 15.3	89.6 ± 4.9	n.s
Koos	77.3 ± 15.5	80.0 ± 5.8	n.s
Oxford	40.0 ± 8.3	43.4 ± 3.2	n.s
KSSc	87.7 ± 15.7	91.0 ± 10.0	n.s
KSSf	83.0 ± 17.4	82.4 ± 13.4	n.s

*FB* fixed bearing, *MB* mobile bearing

**Table 7:** Comparison of clinical outcomes between patients who performed the deep knee lunge and patients who did not

	FB DKL (*n* = 19)	FB No DKL (*n* = 2)	*T* test *P*-value
VAS	2.1 ± 2.6	4.0 ± 5.7	n.s
Womac	82.6 ± 15.3	80.7 ± 24.1	n.s
Koos	75.6 ± 15.4	75.0 ± 15.4	n.s
Oxford	39.3 ± 8.5	34.0 ± 18.4	n.s
KSSc	86.7 ± 16.1	76.0 ± 19.0	n.s
KSSf	81.3 ± 17.4	75.0 ± 25.0	n.s

*FB* fixed bearing, *DKL* deep knee lunge

## Discussion

The main finding of the present study was that posterior-stabilised fixed-bearing TKA design showed statistically significant lower AP translations during STS, lower VV and IE rotations during DKL in comparison to posterior-stabilised mobile-bearing TKA design. Furthermore, analysing the low point kinematics, FB showed a significant lower AP translation of the medial compartment with respect to the MB group, with a higher percentage of medial pivot. The present findings may be attributed to the differences between the two designs. In the MB design, the self-aligning insert increases the contact distribution forces with the femoral component and reduces the stresses transmitted to the tibial post, which maintains a neutral position in relation to the femoral cam. This tolerance increases the magnitude of translations and rotations of the femur compared to the tibial plateau. Moreover, thanks to the capability of the insert to follow the femoral component during rotations [13, 23], MB design should reduce polyethylene wear of the articular surface by decoupling translation and rotation forces between the femur, polyethylene, and the underlying tibial plateau [21]. However, the MB design has been shown to have more backside wear than the FB [25]. Differently, the FB kinematics is partially bound to the medial femoral–tibial compartment in addition to the post-cam system. In a posterior-stabilised TKA, the AP translations of the medial compartment correlates with the patient clinical outcomes and must be taken into account intraoperatively [30]. On the contrary, an over constrained medial femoral tibial compartment reduces the AP translation of the femoral component compared to the tibial plateau and this results in a poor knee function. Furthermore, it has been widely described how fixed-bearing TKAs are affected by eccentric wear of the post [41]. Furthermore, the MB insert should compensate for small rotational defects of the tibial plateau, as in the valgus knees, optimising TKA kinematics, patellar tracking and reducing stresses on polyethylene surface and posterior tibial posts [7, 11, 14, 35]. However, FB design reduces risks of insert dislocation, showing similar clinical outcomes and failure rate compared to MB [39].

Several studies have shown no difference in rotations between FB and MB [3, 12, 38]. In addition, Komistek et al. showed lower rotation of MB than a normal knee [23]. Conversely, other studies reported wider axial rotations and anterior motion of the medial condyle in mobile-bearing than in fixed-bearing TKA [10, 32].

Our findings do not allow to conclude on the superiority of one design over the other. On the one hand, FB design showed a less AP translation of the medial compartment compared to MB design, which could, however, be responsible for increased peak forces on the polyethylene, while at the same time maintaining a wide translation of the lateral compartment; translated, a more pronounced medial pivot behaviour and physiological kinematics. On the other hand, MB design showed higher translations and rotations values than the FB design, while remaining lower than those of a native knee.

In the present study, no differences regarding clinical scores and ROM between the two groups were detected. Although differences were found in the kinematics between the two prosthetic designs, the sample analysed did not have sufficient power to detect potential correlations with clinical outcomes, and this was not among the objectives of the study. However, literature reports no significant differences in the clinical outcomes between posterior-stabilised fixed-bearing and mobile-bearing TKA [19, 31]. The evaluation of larger cohorts would be necessary to obtain sufficient statistical power to correlate a possible influence of kinematics on clinical outcomes, as already done by other authors on different aspect of knee kinematics [22, 34].

The study presents some limitations. First, the low sample size, which is, however, in line with studies assessing TKA kinematics [4, 16, 36]. Second, there is a potential bias due to a significant difference in follow-up time between the two groups. We did not expect influences on kinematics due to such a short time gap, in any case longer than nine months after surgery in both groups. However, two patients in the FB group were unable to perform the DKL for lack of confidence in performing that high demanding motor task. No correlation was found between their clinical outcomes and the rest of the FB cohort, and the investigators did not want to force them to avoid potential falls or knee pain. Third, the deep knee flexion angles (> 100°) have not been investigated because of the difficulties to perform such movements safely in our radiographic set-up. Furthermore, the forces acting on the polyethylene have not been investigated due to the radiotrasparency of such material.

The clinical relevance of the present study is that, despite the kinematic differences emerged, no clinical differences existed between MB and FB designs. Therefore, both designs represent a viable alternative in primary TKA.

## Conclusions

Statistically significant differences have been found between posterior-stabilised fixed-bearing and mobile-bearing TKA in post-operative AP translations, VV rotations and IE rotations of femoral components compared to tibial components in STS and DKL. Furthermore, FB reported a significant higher percentage of medial pivot. Despite this, no clinical differences were detected between the two groups.

Both designs showed stable kinematics and represent a viable alternative in primary TKA.

## Data Availability

Not applicable.
